# A Patient With Granuloma Annulare and Lichen Planus Treated With Apremilast: A Case Report

**DOI:** 10.1155/carm/6883705

**Published:** 2025-08-06

**Authors:** Emre Sarıkaya, Meltem Türkmen, Selcen Kundak, Sümeyye Ekmekci

**Affiliations:** ^1^Department of Dermatology, Izmir Faculty of Medicine, Izmir City Hospital, Health Sciences University, Izmir, Türkiye; ^2^Department of Pathology, Izmir Faculty of Medicine, Izmir City Hospital, Health Sciences University, Izmir, Türkiye

**Keywords:** apremilast, case report, granuloma annulare, lichen planus, treatment

## Abstract

Granuloma annulare (GA) is an inflammatory and granulomatous dermatosis characterized by annular erythematous papules/plaques frequently localized in acral regions. Proinflammatory cytokines such as tumor necrosis factor-alpha (TNF-α) and interferon-gamma (IFN-γ), which are released by T helper 1 (Th1) lymphocytes inducing macrophages, are thought to play a role in its pathogenesis. Lichen planus (LP) is an inflammatory dermatosis characterized by pruritic scaly purple papules, often on the wrists and ankles, and can also affect mucosa, hair, and nails. T-cell-mediated proinflammatory cytokines such as IFN-γ and TNF-α, which are released by macrophages upon Th1 stimulation, have been implicated in the pathogenesis of LP, as in GA. A new treatment option is needed in the treatment of these diseases due to suboptimal results and adverse side-effect profiles with conventional treatments. Apremilast is a phosphodiesterase-4 (PDE4) inhibitor and inhibits the production of various inflammatory mediators such as IFN-γ, TNF-α, IL-2, IL-5, IL-8, IL-12, and leukotriene B4. This molecule has three Food and Drug Administration (FDA) approved indications: moderate to severe plaque psoriasis, psoriatic arthritis, and oral ulcers associated with Behcet's disease. Apremilast exhibits a favorable side-effect profile compared to conventional treatments and is a good treatment option with its ability to reduce cytokines implicated in the pathogenesis of GA and LP. Here, we report the case of a 55-year-old woman in whom apremilast treatment led to an almost complete resolution of her GA and LP.

## 1. Case Report

**Figure 1:**
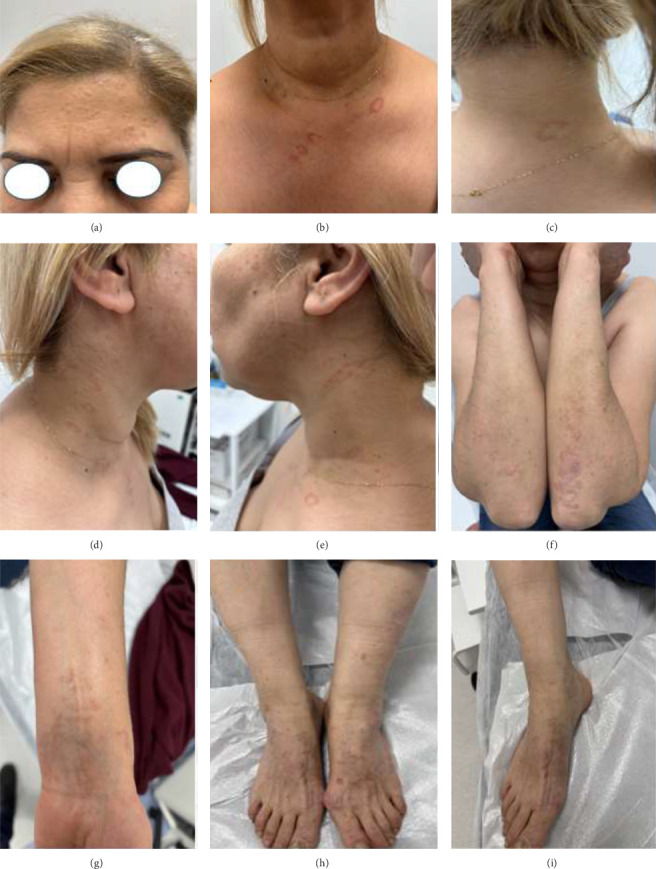
Pre-apremilast clinical presentation.

**Figure 2:**
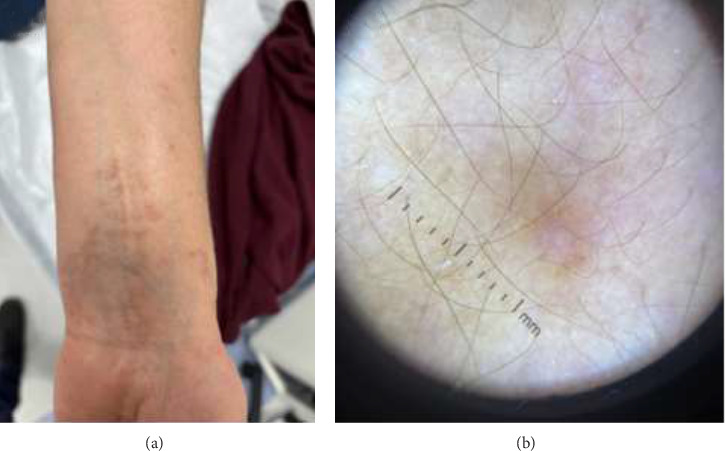
Papule with Wickham striae seen on dermoscopy confirmed lichen planus. (a) Clinical image (b) Dermoscopic image.

A 55-year-old woman diagnosed with lichen planus (LP) by biopsy presented with erythematous plaques on her neck and an itchy skin rash on her upper extremities. She had no history of medication use or allergy. Her past medical history was positive for LP. There was no family history of LP or granuloma annulare (GA). Physical examination revealed erythematous, annular, nonscaly plaques on her neck and anterior chest, flesh-colored papules on her face, pruritic purple papules on the extensor regions of the upper extremities and lower extremities, and purple scaly papular lesions on her medial wrist (Figure 1). Dermoscopy of the purple papule on her medial wrist revealed Wickham striae, confirming LP (Figure 2).

**Figure 3:**
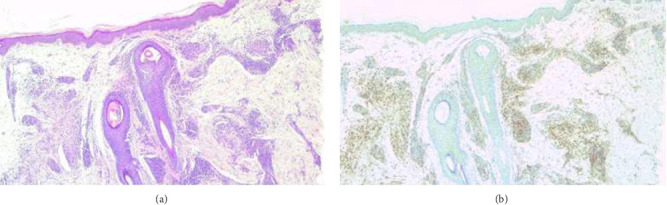
(a) Collagenolytic granulomatous inflammation localized in the dermis (H&E, x100). (b) Positivity of dermal histiocytes with CD68 immunohistochemical stain (DAB, x100).

**Figure 4:**
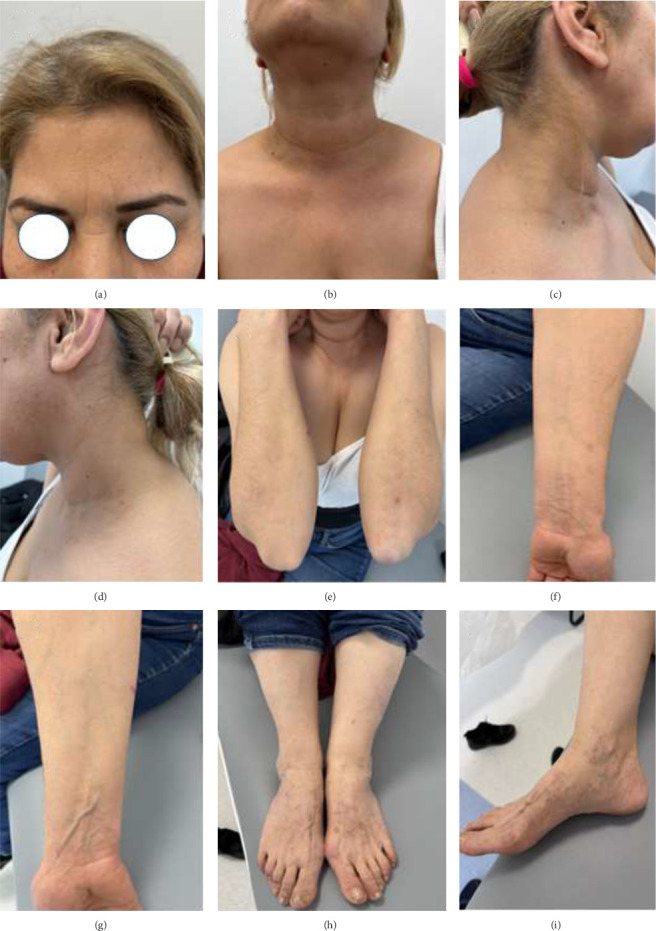
Post-apremilast clinical presentation.

A 4-mm punch biopsy of the lesion on her left arm revealed granulomatous inflammation with collagenolytic areas consistent with GA (Figure 3). Despite treatment with highly potent topical corticosteroids, 0.1% topical calcineurin inhibitor, systemic corticosteroids, and oral isotretinoin, the lesions were persistent and progressive. Apremilast treatment was planned for LP papules resistant to 6 years of treatment and for GA lesions present for 1 year. Other treatments were discontinued, and the patient was started on oral apremilast. The apremilast dose was 10 mg on the first day, with daily increments of 10 mg until a maintenance dose of 30 mg twice daily was reached on Day 6. Six weeks of apremilast treatment resulted in complete regression of the skin-colored papules on the frontal face, the annular erythematous plaques on the neck, and the GA and LP lesions on the distal upper extremities, while the lesions on the distal lower extremities resolved, leaving behind postinflammatory hyperpigmentation (Figure 4). The patient used only apremilast treatment during the 6-week treatment period, the itching complaint completely subsided, and no side effects were observed.

## 2. Discussion

GA is an inflammatory, granulomatous dermatosis of unknown etiology, typically characterized by erythematous annular plaques and flesh-colored papules. It is approximately twice as common in women as in men. In addition to the localized form, which presents with erythematous annular papules/plaques on the dorsum of the hands and feet and is included in the differential diagnosis of tinea, erythema annulare centrifugal, annular LP, necrobiosis lipoidica, lupus, and sarcoidosis, there are also disseminated, subcutaneous, perforating, and patchy forms that can be confused with morphea or mycosis fungoides [1–3]. Although the etiology is not clearly defined, the underlying mechanism is thought to be a type 4 hypersensitivity reaction [4]. Dermal lymphohistiocytic infiltrate and collagen degeneration are typical histopathological findings of GA. T helper 1 (Th1) lymphocytes trigger a delayed-type hypersensitivity reaction by stimulating macrophages to secrete proinflammatory cytokines such as interferon-gamma (IFN-γ) and tumor necrosis factor-alpha (TNF-α) and collagen-degrading enzymes such as matrix metalloproteinase. GA is often difficult to treat due to its refractory nature and lack of evidence-based therapy, and it can lead to an increased psychiatric disease burden, especially in its generalized form [4, 5]. Various molecules, including topical and systemic corticosteroids, calcineurin inhibitors, retinoids, cyclosporine, dapsone, hydroxychloroquine, nicotinamide, allopurinol, hydroxyurea, rifampicin–ofloxacin–minocycline combination, tetracycline, photodynamic therapy, vitamin E, pentoxifylline, zidovudine, TNF-α inhibitors, interferon-α, and antidiabetic agents, have been described in case reports for treatment, but an effective and reliable molecule is needed [6]. Myelosuppression has been reported as a side effect in cases of granuloma ring treated with dapsone [7]. In another case report, a patient with extensive granuloma ring was given 200 mg of hydroxychloroquine twice daily for 6 weeks, which reduced pruritus and healed the lesions by 50%. In our case, 6 weeks of treatment with apremilast resulted in complete resolution of pruritus and almost complete resolution of the lesions [8]. Furthermore, monitoring for eye disease was required with hydroxychloroquine treatment, whereas this was not necessary with apremilast.

LP is a chronic inflammatory dermatosis of unknown etiology that can affect the skin, mucosa, hair, and nails and presents with pruritic hyperkeratotic scaly papules [9]. Although LP is recognized as a chronic T-cell-mediated disease of unknown etiology, a wide variety of contributing factors, such as autoimmunity, microbial and infectious agents, certain drugs and dental materials, nutritional deficiencies, psychological stress, and genetic predisposition, may also influence its pathogenesis [9]. Clinical variants include annular, bullous, hypertrophic, inverse, linear, ulcerative, drug-induced, and lichen planopilaris and can therefore be included in the differential diagnosis of many diseases. T-cell-mediated proinflammatory cytokines such as TNF-α and IFN-γ play a role in its pathogenesis. The histopathology of LP reveals hyperkeratosis, focal increase in the granular layer, keratinization of the basal layer, sawtooth appearance of the epidermis, and band-like lymphocytic infiltrate. I could list some systemic agents used in the treatment of LP, for example, steroids are commonly used for extensive LP, but you cannot maintain a patient on systemic steroids indefinitely [10–12]. Therefore, an optimal treatment option for LP is needed in terms of efficacy, cost, and a safe side-effect profile.

Apremilast is a novel molecule that activates protein kinase A through PDE-4 inhibition, leading to the accumulation of cyclic adenosine monophosphate (cAMP). Increased cAMP inhibits the production of TNF-α, IFN-γ, leukotriene B4, and various proinflammatory cytokines such as IL-2, IL-5, IL-8, and IL-12 [13]. These proinflammatory cytokines play a role in the pathogenesis of GA, LP, psoriasis, and various inflammatory diseases, and case reports in the literature have led to the promotion of apremilast as a treatment option in various chronic inflammatory diseases. Apremilast therapy is relatively easy for patients to use because it does not involve organ toxicity like other conventional systemic therapies, does not require screening for hepatitis virus or tuberculosis, and lacks traditional immunosuppressive properties. However, gastrointestinal side effects can be severe enough to warrant discontinuation of treatment in some patients [14].

In conclusion, GA and LP are chronic, benign inflammatory dermatoses that can cause significant comorbidities. Therefore, patients need an effective and safe treatment option. Apremilast, with its rapid onset of action and favorable side-effect profile, may be an excellent treatment option for these patients, as it was in our patient.

## Data Availability

Data sharing is not applicable to this article as no datasets were generated or analyzed during the current study.

## References

[1] Bolognia J. L., Jorizzo J. L., Rapini R. P. (2008). *Dermatology*.

[2] Mutasim D. F., Bridges A. G. (2000). Patch Granuloma Annulare: Clinicopathologic Study of 6 Patients. *Journal of the American Academy of Dermatology*.

[3] Piette E. W., Rosenbach M. (2016). Granuloma Annulare: Clinical and Histologic Variants, Epidemiology and Genetics. *Journal of the American Academy of Dermatology*.

[4] Kovich O., Burgin S. (2005). Generalized Granuloma Annulare. *Dermatology Online Journal*.

[5] Joshi T. P., Chen V., Dong J.-Li (2022). Psychiatric Comorbidities Associated With Granuloma Annulare: A Case Control Study in the all of Us Database. *Journal of the American Academy of Dermatology*.

[6] Lukács J., Schliemann S., Elsner P. (2015). Treatment of Generalized Granuloma Annulare: A Systematic Review. *Journal of the European Academy of Dermatology and Venereology*.

[7] Hrin M. L., Bashyam A. M., Feldman S. R., Huang W. W. (2022). Oral Dapsone for the Treatment of Generalized Granuloma Annulare: A Retrospective Case Series. *Journal of the American Academy of Dermatology*.

[8] Carlin M. C., Ratz J. L. (1987). A Case of Generalized Granuloma Annulare Responding to Hydroxychloroquine. *Cleveland Clinic Journal of Medicine*.

[9] Saeed S., Choudhury P., Ahmad S. A. (2022). Vitamin D in the Treatment of Oral Lichen Planus: A Systematic Review. *Biomedicines*.

[10] Usatine R. P., Tinitigan M. (2011). Diagnosis and Treatment of Lichen planus. *American Family Physician*.

[11] Lavanya N., Jayanthi P., Rao U. K., Ranganathan K. (2011). Oral Lichen Planus: An Update on Pathogenesis and Treatment. *Journal of Oral and Maxillofacial Pathology*.

[12] Schafer P. H., Parton A., Gandhi A. K. (2010). Apremilast, a Camp phosphodiesterase-4 Inhibitor, Demonstrates Anti-inflammatory Activity In Vitro and in a Model of Psoriasis. *British Journal of Pharmacology*.

[13] Schafer P. H., Parton A., Capone L. (2014). Apremilast is a Selective PDE4 Inhibitor With Regulatory Effects on Innate Immunity. *Cellular Signalling*.

[14] Gooderham M., Papp K. (2015). Selective Phosphodiesterase Inhibitors for Psoriasis: Focus on Apremilast. *BioDrugs*.

